# Antibody-Validated Proteins in Inflamed Islets of Fulminant Type 1 Diabetes Profiled by Laser-Capture Microdissection Followed by Mass Spectrometry

**DOI:** 10.1371/journal.pone.0107664

**Published:** 2014-10-16

**Authors:** Yoriko Nishida, Kaoru Aida, Makoto Kihara, Tetsuro Kobayashi

**Affiliations:** 1 Department of Nursing, Interdisciplinary Graduate School of Medicine and Engineering, University of Yamanashi, Chuo, Yamanashi, Japan; 2 Third Department of Internal Medicine, Interdisciplinary Graduate School of Medicine and Engineering, University of Yamanashi, Chuo, Yamanashi, Japan; 3 Medical ProteoScope Co., Ltd., Yokohama, Kanagawa, Japan; 4 Okinaka Memorial Institute for Medical Research, Tokyo, Japan; University of Siena, Italy

## Abstract

**Background:**

There are no reports of proteomic analyses of inflamed islets in type 1 diabetes.

**Procedures:**

Proteins expressed in the islets of enterovirus-associated fulminant type 1 diabetes (FT1DM) with extensive insulitis were identified by laser-capture microdissection mass spectrometry using formalin-fixed paraffin-embedded pancreatic tissues.

**Results:**

Thirty-eight proteins were identified solely in FT1DM islets, most of which have not been previously linked to type 1 diabetes. Five protein-protein interacting clusters were identified, and the cellular localization of selected proteins was validated immunohistochemically. Migratory activity-related proteins, including plastin-2 (LCP1), moesin (MSN), lamin-B1 (LMNB1), Ras GTPase-activating-like protein (IQGAP1) and others, were identified in CD8^+^ T cells and CD68^+^ macrophages infiltrated to inflamed FT1DM islets. Proteins involved in successive signaling in innate/adaptive immunity were identified, including SAM domain and HD domain-containing protein 1 (SAMHD1), Ras GTPase-activating-like protein (IQGAP1), proteasome activator complex subunit 1 (PSME1), HLA class I histocompatibility antigen (HLA-C), and signal transducer and activator of transcription 1-alpha/beta (STAT1). Angiogenic (thymidine phosphorylase (TYMP)) and anti-angiogenic (tryptophan-tRNA ligase (WARS)) factors were identified in migrating CD8^+^ T cells and CD68^+^ macrophages. Proteins related to virus replication and cell proliferation, including probable ATP-dependent RNA helicase DEAD box helicase 5 (DDX5) and heterogeneous nuclear ribonucleoprotein H (HNRNPH1), were identified. The anti-apoptotic protein T-complex protein 1 subunit epsilon (CCT5), the anti-oxidative enzyme 6-phosphogluconate dehydrogenase (PDG), and the anti-viral and anti-apoptotic proteins serpin B6 (SERPINB6) and heat shock 70 kDa protein1-like (HSPA1L), were identified in FT1DM-affected islet cells.

**Conclusion:**

The identified FT1DM-characterizing proteins include those involved in aggressive beta cell destruction through massive immune cell migration and proteins involved in angiogenesis and islet vasculature bleeding, cell repair, and anti-inflammatory processes. Several target proteins for future type 1 diabetes interventions were identified.

## Introduction

Many cascades related to viral infections and innate and adaptive immunity and beta cell responses are postulated to lead to beta cell dysfunctions in human type 1 diabetes and type 1 diabetic rodent models [1], [2], [3]. Proteins involved in beta cell destruction have been identified based on *in vivo* animal model studies of type 1 diabetes [1], [3], [4], [5]. However, the proteins and mechanisms associated with the destruction or defense of beta cells in human type 1 diabetes have yet to be elucidated. Furthermore, to date there have been no reports of *in situ* protein profiling in human inflamed islets affected by type 1 diabetes (insulitis).

Laser-capture microdissection (LMD) coupled with liquid chromatography (LC)-tandem mass spectrometry (MS) (LMD-LC-MS) is an emerging method useful for profiling proteins *in situ* [6]. We performed a proteomic analysis of formalin-fixed and paraffin-embedded pancreatic islet tissues of enterovirus-associated fulminant type 1 diabetes (FT1DM), a representative subtype of virus-related human type 1 diabetes in which most of the islets are affected by insulitis [7]–[11]. Furthermore, we immunohistochemically validated the localization of the identified proteins in type 1 diabetic islets. An important feature of our findings was how selectively many proteins function in affected islets, including remaining islet cells and infiltrating immune cells. The catalog of profiled proteins and the protein-protein network model presented here provide new insights that will both enhance our understanding of the pathogenesis of virus-induced type 1 diabetes and facilitate development of future interventions. In addition, our LMD-LC-MS method is applicable to routine analysis of pathological specimens of formalin-fixed and paraffin-embedded tissues.

## Research Design and Methods

### Patients and pancreatic tissues

Three pancreatic specimens were obtained at autopsy and fixed with 5% formaldehyde and paraffin-embedded. More than 95% of the islets had extensive mononuclear cell (MNC) infiltration (insulitis) [10], [11]. Clinical profiles of the three autopsied patients with FT1DM were reported elsewhere [10]. Briefly, case 1 was a 14-year-old boy who died from diabetic ketoacidosis following onset of flu-like symptoms 5 days earlier. Case 2 was a 25-year-old man who died from diabetic ketoacidosis following sudden onset of nausea and symptoms of epigastric pain 2 days earlier. Case 3 was a 29-year-old man who died from diabetic ketoacidosis following onset of slight fever, nausea, and vomiting 2 days earlier.

### Non-diabetic control subjects

Pancreatic tissues from five autopsied non-diabetic men (22±4 years of age, mean ± SD, range: 18–28 years of age) were used as non-diabetic controls.

### Immunostaining of the pancreas

Methods for immunohistochemical analyses were reported previously [10], [11]. The primary antibodies used in this study are listed in Table S1.

### LMD and protein extraction

Serial 10-µm-thick sections were prepared from pancreas blocks and attached onto DIRECTOR™ slides (Expression Pathology, Rockville, MD). Every fifth section was stained with anti-insulin antibody (DAKO, Carpinteria, CA) as described elsewhere [10], [11]. For LMD, the sections were de-paraffinized twice with xylene for 5 min, rehydrated with graded ethanol solutions and distilled water and then stained with hematoxylin alone. Stained uncovered slides were air-dried and 6.4∼6.8 mm^2^ islets area (1,700∼2,700 islets) were collected in a 0.2-mL low-binding plastic tube using a Leica LMD7000 (Leica Microsystems GmbH, Ernst-Leitz-Strasse, Wetzlar, Germany). Protein extraction was performed using a Liquid Tissue MS Protein Prep Kit (Expression Pathology) according to the manufacturer's protocol. Because tissues from FT1DM cases were scarce, islets obtained from three FT1DM pancreata by LMD were combined and analyzed using mass spectrometry.

### LC-MS

LC-MS analysis of digested samples was carried out essentially as described previously [6]. We used a reversed-phase liquid chromatography (RP-LC) system interfaced with an LTQ-Orbitrap hybrid mass spectrometer (Thermo Fisher Scientific, Bremen, Germany) equipped with a nano-electrospray source (AMR, Tokyo, Japan). The RP-LC system (Paradigm MS4, Michrom BioResources, Auburn, CA) consisted of an L-column Micro Trap (0.3×5.0 mm) and a capillary separation column (0.1×150 mm L-column Micro packed with reversed-phase L-C18 gel particles of 3 µm in diameter and 12-nm pore size [CERI, Tokyo, Japan]) fitted with an emitter tip (FortisTip, 20-µm ID and 150-µm OD with a perfluoropolymer-coated blunt end, OmniSeparo-TJ, Hyogo, Japan). An autosampler (HTC-PAL, CTC Analytics, Zwingen, Switzerland) was used to load aliquots of samples onto the trap, which then was washed with solvent A (98% distilled water with 2% acetonitrile and 0.1% formic acid) for concentrating peptides on the trap and desalting. Subsequently, the trap was connected in series to the analytical column and the columns were eluted for 100 min at a flow rate of 0.3 µL/min with a linear gradient from 5% solvent B (10% distilled water and 90% acetonitrile containing 0.1% formic acid) to 45% B, then from 45% B to 90% B over 5 min, maintaining at 90% B for 5 min, then from 90% B to 5% B over 0.01 min, followed by re-equilibration with 5% B for 9 min.

The LTQ was operated in the data-dependent MS/MS mode to automatically acquire up to three successive MS/MS scans in the centroid mode. The three most intense precursor ions for these MS/MS scans could be selected from a high-resolution MS spectrum (survey scan) previously acquired by the Orbitrap during a predefined short time window in the profile mode at a resolution of 30,000 in the *m/z* range 450 to 1,800. The sets of acquired high-resolution MS and MS/MS peptide spectra were converted to single data files and merged into Mascot generic format files for database searching.

### Database searching and semi-quantification with spectral counting

All MS/MS data were searched against the UniProt/Swiss-Prot (release 2012_03) database using Mascot (version 2.2.06, Matrix Science, London, UK), in which the peptide and fragment mass tolerances were 10 ppm and 0.8 Da, respectively, and up to two missed cleavages were allowed for errors in trypsin specificity. For variable peptide modifications, methionine oxidation and formylation of lysine, arginine, and N-terminal amino acids were taken into account. Reported results were obtained from triplicate LC-MS runs for each sample with all peptide hits included. Unique peptides and proteins were identified by the following proteomics guidelines. Mascot search results were processed through Scaffold software (version 3.3.3, Proteome Software, Portland, OR) for gene ontology analyses and validation of MS/MS-based peptide and protein identifications. Peptide identifications were accepted if they could be established at a Scaffold peptide probability of >95%. Protein identifications were accepted if they could be established at a Scaffold protein probability of >99% and contained at least two identified peptides. Identified proteins were also analyzed in terms of putative functional association networks using the STRING 9.01 Server (http://www.string-db.org).

### Ethical considerations

The Ethics Committee of the University of Yamanashi approved all of the procedures performed in this study. Witten informed consent was obtained from the next of kin or parents/guardians on behalf of the children or autopsied cases. The informed consent was written on the form and kept in the medical records. The Ethics Committee of the University of Yamanashi approved the consent procedures.

### Statistical analysis

Fisher's exact test was used to compare the frequencies of specific immunostaining results between FT1DM-affected and non-diabetic control pancreata.

## Results and Discussion

### Proteins identified by LMD-LC-MS and the protein profile of inflamed FT1DM pancreas tissue

We identified a total of 300 different proteins. A total of 193 proteins were identified in the islet mixture from the three FT1DM patients, and 262 proteins were identified in the islets of at least one of the control patients (Table 1 and Tables S2 and S3). Overall, 38 of the 300 proteins identified (12.7%) (Table 1) were found only in the FT1DM islets, 107 proteins (35.7%) (Table S3) were found only in the control islets, and 155 proteins (51.7%) (Table S2) were found in both control and FT1DM-affected islets. Most proteins have not been previously implicated as being involved in type 1 diabetes (Table 1). The proteome of the control islets included insulin, glucagon, and proteins associated with glycolysis/gluconeogenesis, oxidative phosphorylation, ribosomes, and secretory granules (Tables S2 and S3). Glucagon was identified in FT1DM-affected islets but insulin was not, which was in agreement with our previous pathological studies [10], [11].

**Table 1:** Proteins identified only in islets affected by fulminant type 1 diabetes.

	Accession number	Entry name	Protein names	Gene names	Molecular weight
1	Q71U36	TBA1A_HUMAN	Tubulin alpha-1A chain	TUBA1A	50 kDa
2	P52272	HNRPM_HUMAN	Heterogeneous nuclear ribonucleoprotein M	HNRNPM	78 kDa
3	P13796	PLSL_HUMAN	Plastin-2	LCP1	70 kDa
4	P11678	PERE_HUMAN	Eosinophil peroxidase	EPX	81 kDa
5	P23381	SYWC_HUMAN	Tryptophan–tRNA ligase, cytoplasmic	WARS	53 kDa
6	P42224	STAT1_HUMAN	Signal transducer and activator of transcription 1-alpha/beta	STAT1	87 kDa
7	P30504	1C04_HUMAN	HLA class I histocompatibility antigen, Cw-4 alpha chain	HLA-C	41 kDa
8	P19971	TYPH_HUMAN	Thymidine phosphorylase	TYMP	50 kDa
9	P61313	RL15_HUMAN	60S ribosomal protein L15	RPL15	24 kDa
10	O60814	H2B1K_HUMAN	Histone H2B type 1-K	HIST1H2BK	14 kDa
11	P28838	AMPL_HUMAN	Cytosol aminopeptidase	LAP3	56 kDa
12	P46940	IQGA1_HUMAN	Ras GTPase-activating-like protein IQGAP1	IQGAP1	189 kDa
13	P61158	ARP3_HUMAN	Actin-related protein 3	ACTR3	47 kDa
14	Q06323	PSME1_HUMAN	Proteasome activator complex subunit 1	PSME1	29 kDa
15	O14950	ML12B_HUMAN	Myosin regulatory light chain 12B	MYL12B	20 kDa
16	|P26038	MOES_HUMAN	Moesin	MSN	68 kDa
17	O60506	HNRPQ_HUMAN	Heterogeneous nuclear ribonucleoprotein Q	SYNCRIP	70 kDa
18	P48643	TCPE_HUMAN	T-complex protein 1 subunit epsilon	CCT5	60 kDa
19	P31946	1433B_HUMAN	14-3-3 protein beta/alpha	YWHAB	28 kDa
20	Q9UL46	PSME2_HUMAN	Proteasome activator complex subunit 2	PSME2	27 kDa
21	P10412	H14_HUMAN	Histone H1.4	HIST1H1E	22 kDa
22	P17844	DDX5_HUMAN	Probable ATP-dependent RNA helicase DDX5	DDX5	69 kDa
23	P35237	SPB6_HUMAN	Serpin B6	SERPINB6	43 kDa
24	Q9BQE5	APOL2_HUMAN	Apolipoprotein L2	APOL2	37 kDa
25	P08729	K2C7_HUMAN	Keratin, type II cytoskeletal 7	KRT7	51 kDa
26	P20700	LMNB1_HUMAN	Lamin-B1	LMNB1	66 kDa
27	P62847	RS24_HUMAN	40S ribosomal protein S24	RPS24	15 kDa
28	Q9Y3Z3	SAMH1_HUMAN	SAM domain and HD domain-containing protein 1	SAMHD1	72 kDa
29	O60361	NDK8_HUMAN	Putative nucleoside diphosphate kinase	NME2P1	16 kDa
30	O95154	ARK73_HUMAN	Aflatoxin B1 aldehyde reductase member 3	AKR7A3	37 kDa
31	P13473	LAMP2_HUMAN	Lysosome-associated membrane glycoprotein 2	LAMP2	45 kDa
32	P21333	FLNA_HUMAN	Filamin-A	FLNA	281 kDa
33	P31943	HNRH1_HUMAN	Heterogeneous nuclear ribonucleoprotein H	HNRNPH1	49 kDa
34	P52209	6PGD_HUMAN	6-phosphogluconate dehydrogenase, decarboxylating	PGD	53 kDa
35	P59998	ARPC4_HUMAN	Actin-related protein 2/3 complex subunit 4	ARPC4	20 kDa
36	P16401	H15_HUMAN	Histone H1.5	HIST1H1B	23 kDa
37	P34931	HS71L_HUMAN	Heat shock 70 kDa protein 1-like	HSPA1L	70 kDa
38	O60763	USO1_HUMAN	General vesicular transport factor p115	USO1	108 kDa

### LMD-LC-MS-identified and antibody-validated proteins in FT1DM-affected islets and protein-protein interactions

In FT1DM, enterovirus infection induces innate immune responses, activates the CXCL10-CXCR3 axis, and induces massive infiltration of autoreactive T cells, dendritic cells, and macrophages, resulting in subsequent severe and rapid beta cell destruction [10], [11]. We therefore sought to identify proteins that promote beta cell destruction *in situ* using LMD-LC-MS, and we immunohistochemically validated the presence and cellular localization of some of the candidate proteins, as described below.

**Figure 1:**
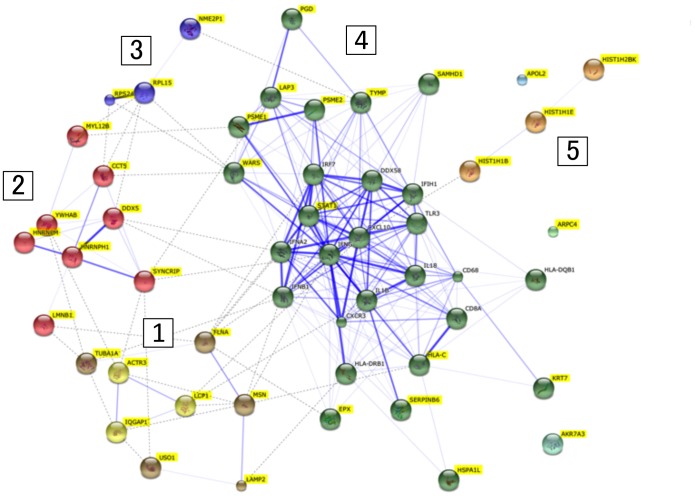
Protein-protein interaction networks involving 38 islet proteins unique to FT1DM-affected tissue that were identified by LMD-LC-MS in this study (yellow labeled) and proteins previously identified immunohistochemically in inflamed FT1DM-affected islets [10], [11] and classified using STRING (http://www.string-db.org). The five clusters are indicated by numerals. The width of the edges depends on the confidence score for each protein association as determined by STRING analysis.

**Figure 2:**
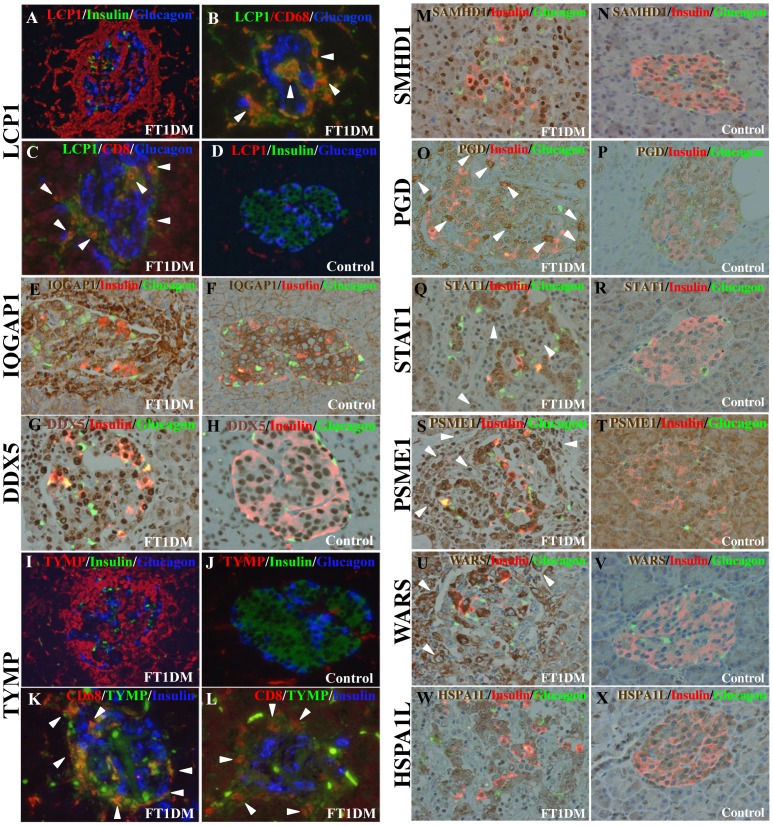
Immunohistochemical validation of the presence of LMD-LC-MS-identified proteins in FT1DM-affected islets. (A)–(D), Triple immunostaining for plastin-2 (LCP1) expression in FT1DM-affected pancreas (A)–(C) and non-diabetic control (D) tissues. (A), Triple immunostaining for plastin-2 (LCP1) in FT1DM-affected pancreas. LCP1 (red) was over-expressed in mononuclear cells (MNCs) that aggressively infiltrated to or around the islets (green: insulin, blue: glucagon). (B), Merged image of triple immunostaining for LCP1 (green), CD68^+^ macrophages (red), and glucagon (blue). Many MNCs are positive for both CD68 and LCP1 and appear yellow (arrowheads). (C), Merged image of triple immunostaining for LCP1 (green), CD8 (red), and glucagon (blue). Many MNCs are positive for both CD8 and LCP1 and appear yellow (arrowheads). (D), Merged image of triple immunostaining for LCP1 (red), insulin (green), and glucagon (blue) in non-diabetic pancreas tissue. Few cells are positive for LCP1. (E)–(F), Expression of Ras GTPase-activating-like protein (IQGAP1) in the islets and MNCs in FT1DM-affected (E) and non-diabetic (F) islets. IQGAP1 (brown) was highly expressed in infiltrating MNCs and some FT1DM islet cells (insulin: red, glucagon: green). (G)–(H), DEAD box helicase5 (DDX5) expression in FT1DM-affected (G) and control (H) pancreas. DDX5 (brown) was hyper-expressed in the nucleus and cytoplasm of all subsets of islet cells, including beta cells (red) and alpha cells (green). Weak expression of DDX5 was observed in the nucleus of non-diabetic pancreas cells (H). (I)–(L), Expression of thymidine phosphorylase (TYMP) in FT1DM-affected (I) and control pancreas (J) tissues. TYMP was over-expressed in MNCs infiltrated to the islets in FT1DM tissue (I). No expression of TYMP was observed in non-diabetic control pancreas tissue (J). Triple immunostaining of FT1DM pancreatic tissue for TYMP (green), CD68^+^ (red), and insulin (blue). Merged image (K) shows that TYMP is expressed on CD68^+^ macrophages and appears yellow (arrowheads). Triple immunostaining of FT1DM-affected pancreatic tissue for TYMP (green), CD8^+^ (red), and insulin (blue). Merged image (L) shows that TYMP is localized on CD8^+^ T cells (arrowheads). (M)–(N), Expression of SAM domain and HD domain-containing protein 1 (SAMHD1) in FT1DM-affected pancreas (M) and control pancreas (N) tissues. Hyper-expression of SAMHD1 (brown) in the nucleus and cytoplasm of islet beta cells (red), alpha cells (green), and infiltrating MNCs is shown. No expression of SAMHD1 was observed in non-diabetic control pancreas tissue (N). (O)–(P), 6-Phosphogluconate dehydrogenase, decarboxylating (PGD) expression in FT1DM-affected pancreas tissue (O). PDG (brown) was over-expressed in the cytoplasm of islet-cells, and non-islet cells (arrowheads). PDG was only faintly expressed in the cytoplasm of non-diabetic islet cells (P). (Q)–(R), Signal transducer and activator of transcription-1 alpha/beta (STAT1) expression in FT1DM-affected pancreas (Q) and control pancreas (R) tissues. STAT1 was over-expressed in the cytoplasm and nucleus of islet beta cells (red), alpha cells (green), and MNCs (arrowheads). No staining for STAT1 was observed in control islet tissue (R). (S)–(T), Proteasome activator complex subunit 1 (PSME1, PA28a) expression in FT1DM-affected pancreas (S) and non-diabetic pancreas (T) tissues. PSME1 (brown) was over-expressed in the nucleus and cytoplasm of beta cells (red), alpha cells (green), and infiltrating MNCs (arrowheads) (S). PSME1 was not expressed in non-diabetic control pancreas tissue (T). (U)–(V), Tryptophanyl-tRNA synthetase (WARS) expression in the islets of FT1DM-affected (U) and non-diabetic control (V) pancreas tissues. Merged image shows expression of WARS (brown) in beta cells (red) and MNCs (arrowheads) in FT1DM-affected tissue (U). No staining of WARS was observed in non-diabetic control pancreas tissue (V). (W)–(X), Heat shock protein 70 kDa protein 1-like (HSPA1L) expression in the islets of FT1DM-affected (W) and non-diabetic pancreas (X) tissues. Strong expression of HSPA1L (brown) was observed in beta cells (red), alpha cells (green), and other subsets of endocrine cells (W). Weak expression of HSPA1L was observed in non-diabetic control islets (X). Positive staining for each identified protein was significantly more frequent (*P* = 0.048) in FT1DM-affected islets than control islets.

**Figure 3:**
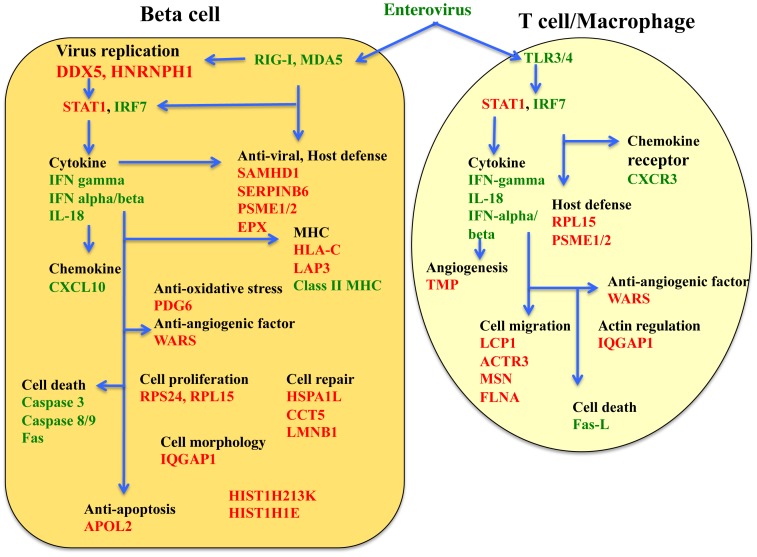
The location and putative function of proteins unique to FT1DM as identified by LMD-LC-MS in this study (red characters) and proteins previously identified by immunohistochemistry (green characters) in FT1DM-affected pancreas tissue [10], [11].

A protein-protein interaction network consisting of 38 proteins unique to FT1DM, and proteins previously identified immunohistochemically in inflamed FT1DM [10], [11], was constructed for FT1DM-affected pancreas tissue. As shown in Figure 1, there appear to be five protein-protein interacting clusters as determined by STRING analyses. Figure 2 shows the cellular locations of the selected proteins in the islets, and Figure 3 summarizes the putative functions and locations of the proteins in FT1DM-affected islets.

### *Cluster 1* (LCP1, ACTR3, IQGAP1, MSN, FLNA, LMNB1, USO1/p115)

Cluster 1 includes proteins that interact with the MNC cytoskeleton and are involved in activation, polarization and infiltration of these cells to the islets. Plastin-2 (LCP1) was highly expressed on CD8^+^ T cells and CD68^+^ macrophages that aggressively infiltrated to or around the islets in FT1DM tissue (Figure 2A–D). LCP1 has been implicated in cell migration [12], [13]. Chemokine binding to the receptor results in phosphorylation of LCP1, which induces movement of the F-actin cytoskeleton to the leading edge of the T cell and subsequent activation of cellular polarization and migration of the chemokine-stimulated T lymphocytes [12], [13]. Thus, aggressive homing of activated CD8^+^ T cells to affected islets in response to CXCL10 chemokine stimulation in FT1DM can be explained by the increased expression of LCP1.

Actin-related protein 3 (ACTR3) was over-expressed in the cytoplasm and nucleus of MNCs infiltrated to the islets in FT1DM-affected pancreas tissue. ACTR3 is a major constituent of the actin-related protein (ARP) 2/3 complex and is postulated to be located at the cell surface and to play an essential role in cell motility [14].

Ras GTPase-activating-like protein (IQGAP1) was highly expressed in infiltrating MNCs, islet beta cells, and islet non-beta cells (Figure 2E). In non-diabetic controls, IQGAP1 was weakly expressed in the islet cells (Figure 2F). IQGAP1 regulates cell morphology and motility through interaction with components of the cytoskeleton (i.e., ACTR3 in cluster 1) as well as cell adhesion molecules and several signaling molecules [15], [16]. Similar to LCP1, IQGAP1 expression on MNCs contributes to the aggressive translocation of MNCs to beta cells in FT1DM.

Moesin (MSN) was hyper-expressed in the cytoplasm of infiltrating CD8^+^ T cells and CD68^+^ macrophages. Moesin plays crucial roles in mediating cell-cell contact between lymphocytes and movement of cells to the target site [14], [17]. This effect on cell-cell contact and movement is mediated by the T cell receptor and may play a role in the aggressive infiltration of cells that is characteristically observed in islets affected by FT1DM [7]–[11].

Filamin A (FLNA), an actin filament cross-linking protein similar to LCP-1 and initially identified in macrophages, plays a central role in mechanotransduction [18] and is also involved in aggressive T cell/macrophage translocation and infiltration into islets.

Lamin B-1 (LMNB1) is expressed in the nuclei of MNCs that have infiltrated into islets in FT1DM-affected pancreas tissue. The lamins play important roles in providing mechanical support and shape to the nucleus and in regulating many nuclear functions, including DNA replication, RNA polymerase II transcription, DNA repair, mitotic spindle formation, response to oxidative stress and chromosome positioning [19].

General vesicular transporter factor p115 (USO1, p115) was densely stained in alpha-cells in FT1DM-affected pancreas tissue. USO1 associates with gamma-tubulin and plays a role in Golgi structure and mitosis progression [20].

### *Cluster 2* (DDX5, HNRNPH1, CCT5)

Cluster 2 included several proteins involved in virus replication. Probable ATP-dependent RNA helicase DEAD box helicase 5 (DDX5) was over-expressed in the nucleus and cytoplasm of all subsets of islet cells affected by FT1DM (Figure 2G). In the nucleus of non-diabetic islet cells, staining of DDX5 was faint (Figure 2H). DDX5 is a member of the Asp-Glu-Ala-Asp (DEAD) box family of RNA helicases and interacts directly with viral proteins and is involved in virus production [21], [22]. These findings are in agreement with our previous finding that enteroviruses are capable of infecting and replicating in all subsets of endocrine cells [10], [11].

Heterogeneous nuclear ribonucleoprotein H (HNRNPH1) was over-expressed in the nucleus of FT1DM-affected islet cells. HNRNPH1 is also involved in virus replication [23].

T-complex protein 1 subunit epsilon (CCT5) chaperone protein was also over-expressed in the islet cells of FT1DM-affected pancreas. CCT5 is a chaperone protein known to play a role in the proper folding of actin and tubulin [24].

### *Cluster 3* (RPS24, RPL15)

Cluster 3 included several ribosomal proteins. Ribosomal proteins are vital for cell proliferation, apoptosis and survival processes [25]. 40S ribosomal protein S24 (RPS24) and 60S ribosomal protein L15 (RPL15) were identified as part of cluster 3. RPL15 also plays a role in innate immune responses stimulated by type I IFN [26].

### *Cluster 4* (TYMP, SAMHD1, SERPINB6, PGD, STAT1, HLA-C, PSME1/PA28a, PSME2/PA28b, WARS, HSPA1L/HSP70T, EPX, LAP3)

Cluster 4 included several proteins related to viral infection, innate/adaptive immunity, and successive signal transduction. Thymidine phosphorylase (TYMP) was highly expressed in CD68^+^ macrophages and CD8^+^ T cells infiltrating to FT1DM-affected islets (Figure 2I, J, K, L). TYMP is a well-known mediator induced by pro-inflammatory and pro-angiogenic conditions [27]–[29]. However, there are no reports regarding the involvement of TYMP in islet inflammation and beta cell destruction. In many types of cancers, TYMP is expressed in the cancer cells and by infiltrating macrophages [27]. TYMP is assumed to contribute to angiogenesis by accelerating the migration of endothelial cells. Expression of TYMP is induced by many inflammatory mediators including IFN-gamma, IFN-alpha, TNF-alpha, and IL-1 beta [27], [28]. The JAK-STAT signaling pathway, probably through STAT1 (see below), mediates IFN-gamma-induced activation of macrophages/T cells and exhibits angiogenic effects, such as migration to islet cell clusters and vascular neogenesis with bleeding, which are characteristic pathological feature of FT1DM. This in turn leads to aggressive macrophages/T cell infiltration to the islets and massive destruction of beta cells. TYMP is a target of manipulation in oncology, suggesting that it could serve as a target for interventional applications of anti-cancer agents [29] to the treatment of type 1 diabetes.

SAM domain and HD domain-containing protein 1 (SAMHD1) was highly expressed in the nucleus and cytoplasm of islet beta cells, non-beta cells, and infiltrating MNCs in FT1DM-affected islets (Figure 2M, N). SAMHD1 was initially isolated as an IFN-gamma-induced factor in dendritic cells, and viral infection induces its expression [30]. Mutations in SAMHD1 are associated with Aicardi-Goutieres syndrome, which resembles a congenital viral infection [31].

Serpin B6 (SERPINB6) was strongly expressed both in islet cells and exocrine cells in FT1DM-affected tissues but was only weakly expressed in non-diabetic control tissues. SERPINB6 is a member of the serpin (serine protease inhibitor) superfamily. Serpins are structurally related proteins that inhibit the activity of serine proteases involved in a diverse array of processes, such as inflammation, apoptosis, coagulation, and tumorigenesis [32]. SERPINB6 also inhibits microbial and viral proteases [33]. SERPINB6 expression is induced by virus infection-associated IFN-gamma production and confers resistance to cytotoxic T cells and NK cells [33]. In the islets of non-obese diabetic (NOD) mice, the expression of mRNAs of several serpin family members was shown to be highly upregulated. Treatment with the serpin family member alpha1-antitrypsin reduces insulitis and prevents diabetes in NOD mice [34]. Clinical trials evaluating alpha-antitrypsin are underway (http://clinicaltrials.gov/ct2/show/NCT01319331).

6-Phosphogluconate dehydrogenase, decarboxylating (PGD), a key enzyme of the pentose phosphate pathway that is responsive to increased oxidative stress [35], was over-expressed in islet-cells and non-islet cells (Figure 2O). PGD over-expression is indicative of compensatory responses to increased oxidant activity in the inflamed milieu of FT1DM.

Signal transducer and activator of transcription 1-alpha/beta (STAT1) was highly expressed in islet endocrine cells and MNCs in FT1DM tissue (Figure 2Q, R). STAT1 is a key transcription factor of antiviral responses and inflammation. STAT1 is activated upon phosphorylation by various ligands (i.e., IFN-gamma, IFN-alpha). Many functions of IFN-gamma have been ascribed to direct STAT1-mediated induction of a number of effector genes, including those encoding various immune-modulatory proteins (i.e., MHC class I and MHC class II), antiviral proteins (i.e., double-stranded RNA-activated protein kinase), antimicrobial proteins (i.e., iNOS), apoptosis-inducing proteins (i.e., IRF-1, Fas/Fas ligand), and cytokines (i.e., MIG, CXCL10, ICAM-1, MCP-1) [36].

HLA class I histocompatibility antigen-C (HLA-C) interacts with cytotoxic T cells to activate them. In enterovirus-induced FT1DM, HLA class I molecules are over-expressed in islet cells, and beta cells secrete CXCL10 [10]. CXCL10 expression may recruit islet-reactive T cells [37]. It was also reported that peripheral T cells from some FT1DM patients are reactive against self-antigens such as GAD and insulin-B-9-23 [38], [39]. These findings suggest a crucial role for HLA-C in both rapid beta cell destruction and the regulation of virus exclusion.

Proteasome activator complex subunit 1 (PSME1, PA28a) and proteasome activator complex subunit 2 (PSME2, PA28b) were over-expressed in the nucleus and cytoplasm of islet beta cells, non-beta cells, and infiltrating MNCs in FT1DM-affected islets (Figure 2S). Proteasomes are constitutively expressed in almost all cells and function in cleaving peptides in an ATP/ubiquitin-dependent process in a non-lysosomal pathway. PSME 1 and 2 are constituents of the immunoproteasome, which is an alternative type of proteasome that plays a central role at the interface between the innate and adaptive immune responses. Immunoproteasomes are constitutively expressed in immunity-related cells and somatic cells, including islet beta cells, after stimulation with IFN-gamma and IFN-beta [40]. The immunoproteasome degrades peptides in a ubiquitin-independent manner and is exclusively connected with the adaptive immune response and increased MHC class I antigen presentation. In addition, the immunoproteasome maintains protein homeostasis against IFN-induced oxidative stress [40]. We previously reported strong expression of IFN-gamma and IFN-beta in FT1DM-affected islet cells [11]. In response to IFN-gamma-induced oxidative stress, PSME1 and 2 will act to maintain cell homeostasis and MHC class I expression.

Tryptophanyl-tRNA synthetase (WARS) was strongly expressed in the beta cells and in MNCs infiltrating the islets (Figure 2U) in FT1DM-affected tissue. While WARS catalyzes the aminoacylation of tRNAs with tryptophan, they also play roles in splicing, apoptosis, viral assembly, and in regulating transcription and translation [41]. WARS expression is strongly induced by IFN-gamma. WARS is secreted by various cell types, and this protein exhibits potent anti-angiogenic activity through inhibition of the expression in endothelial cells of genes encoding proteins associated with angiogenesis, such as Akt and NO synthetase [42]. Negative regulation of angiogenesis induced by viral infection is important in reducing the inflammatory angiogenic response in FT1DM.

Heat shock 70 kDa protein1-like (HSPA1L or HSP70T) was over-expressed in the cytoplasm of all islet cell subsets (Figure 2W). HSPA1L is an isoform of 70 kDa heat shock protein. In conjunction with other heat shock proteins, HSPA1L is also annotated as a protein involved in type 1 diabetes [43]. HSPA1L exhibits chaperone activity in the remaining islet cells in FT1DM-affected pancreas, which is in agreement with findings indicating that all subsets of islet endocrine cell are involved in the inflamed milieu and are affected by the degenerative processes in FT1DM [10], [11].

Eosinophil peroxidase (EPX) is an enzyme that is thought to be involved in host defense in inflammation.

Leucine aminopeptidase 3 (LAP3) was over-expressed in both MNCs and the cytoplasm of FT1DM-affected islet cells. LAP3 expression is induced by IFN-gamma. LAP3 cleaves a single residue from the amino terminus of a peptide to promote antigen presentation by MHC class I molecules.

### *Cluster 5* (HIST1H213K, HIST1H1E, HIST1H1B)

Histone H2B type 1-K (HIST1H213K), histone H1.4 (HIST1H1E), and histone H1.5 (HIST1H1B) are core components of the nucleosome. FoxP3 interacts with HIST1H1B to alter its binding to target genes in order to modulate their expression and to program the regulatory T cell (Treg).

### Non-clustered proteins (APOL2)

Apolipoprotein L2 (APOL2) was over-expressed in islet endocrine cells. APOL2 is stimulated by IFN-gamma and exhibits anti-apoptotic activity in IFN-gamma-induced cytotoxicity [44].

## Conclusion

The proteins we profiled in FT1DM-affected islets are primarily expressed in islet endocrine cells and/or CD8^+^ T cells and CD68^+^ macrophages infiltrating the islets. We detected expression in islet cells of viral replication-associated proteins typically induced by virus infection and proteins for which expression is induced by IFN-gamma. These proteins exert their major effects through the JAK-STAT1 signaling cascade, which leads to activation of innate/adaptive immunity, up-regulation of angiogenesis and anti-angiogenesis factors, and initiation of cell repair mechanisms through chaperone activity. In CD8^+^ T cells and CD68^+^ macrophages in the islets, proteins involved in cell motility and migration to target islets are over-expressed. We hypothesize that through immunological mechanisms and angiogenesis with bleeding in the pancreas, some of the identified proteins play crucial roles in the aggressive beta cell destruction that is a characteristic pathological feature of FT1DM. Other molecules we identified have antiviral, cell repair, and anti-inflammatory actions. Several proteins that may prove useful in future vaccination or chemical interventions to treat type 1 diabetes were also identified.

We could not identify some proteins that were previously detected immunohistochemically, including enterovirus capsid protein (VP1), RIG-I, and MDA5 [10], [11]. The very low amount of protein present in samples from FT1DM-affected islets may explain our inability to identify these proteins in the present study. Proteins present in crude samples at the femtomole (10^−15^) level can be detected using LCM-LC-MS, whereas immunohistochemical staining enables detection of molecules present at the attomole (10^−18^) level, depending on the antisera used.

## Supporting Information

Table S1 **Antibodies used in this study.**
(DOCX)

Table S2 **Proteins identified in both islets affected by fulminant type 1 diabetes and in non-diabetic control pancreatic islets.**
(DOCX)

Table S3 **Proteins identified only in non-diabetic control pancreatic islets.**
(DOCX)
