# Supplementary material for: Antibody-Validated Proteins in Inflamed Islets of Fulminant Type 1 Diabetes Profiled by Laser-Capture Microdissection Followed by Mass Spectrometry
Source: PLoS One. 2014 Oct 16;9(10):e107664. doi: 10.1371/journal.pone.0107664 (PMC4199548; doi:10.1371/journal.pone.0107664)
Supplement: Table S1 — Antibodies used in this study. (DOCX) [file pone.0107664.s001.docx]

**Supporting Information Table S1.**

| Antigen | Species | Clone | Source |
| --- | --- | --- | --- |
| Insulin | Guinea pig | - | Dako, Carpinteria, CA |
| Insulin | Mouse | K36aC10 | Sigma, St. Louis, MO |
| Glucagon | Rabbit | - | Dako, Carpinteria, CA |
| Glucagon | Guinea pig |  | Linko, St. Charles, MD |
| Glucagon | Mouse | K79bB10 | Abcam, Cambridge, UK |
| Somatostatin | Mouse | SOM-018 | GeneTex, Irvine, CA, |
| Plastin-2 (LCP1) | Rabbit | EPR4278 | Abcam, Cambridge, UK |
| Actin-related protein 3 (ATCR3) | Mouse |  | Abcam, Cambridge, UK |
| Ras GTPase-activating-like protein (IQGAP1) | Rabbit | EPR5221 | Abcam, Cambridge, UK |
| Moesin | Rabbit | EPR2428(2) | Abcam, Cambridge, UK |
| Lamin B-1 (LMNB1) | Rabbit | EPR8985(B) | Abcam, Cambridge, UK |
| General vesicular transporter factor p115 (USO1, p115) | Rabbit |  | Abcam, Cambridge, UK |
| Probable ATP-dependent RNA helicase DEAD box helicase 5 (DDX5) | Rabbit | EPR7239 | Abcam, Cambridge, UK |
| Heterogeneous nuclear ribonucleoprotein H (HNRNPH1) | Rabbit |  | Abcam, Cambridge, UK |
| T-complex protein 1 subunit epsilon (CCT5) | Rabbit | EPR7562 | Abcam, Cambridge, UK |
| Thymidine phosphrylase (TYMP) | Rabbit |  | Novus Biologicals, Littleton, CO |
|  |  |  | (Continued) |
| **Supplementary Table 1. (Continued)** |  |  |  |
| SAM domain and HD domain 1 (SAMHD1) | Mouse | 1A1 | Abcam, Cambridge, UK |
| SerpinB6 (SERPINB6) | mouse | 2F8 | Abcam, Cambridge, UK |
| 6-phosphogluconate dehydrogenase (PGD) | Rabbit | EPR6565 | Abcam, Cambridge, UK |
| Signal transducer and activator of transcrition 1- alpha/beta (STAT1) | Rabbit |  | Abcam, Cambridge, UK |
| MHC class I histocompatibility antigen-C (HLA-C) | Mouse | EMR8-5 | Hokudo, Sapporo, Japan |
| Proteasome activator complex subunit 1 (PSME1, PA28A) | Rabbit | EPR10968(B) | Abcam, Cambridge, UK |
| Tryptophanyl-tRNA synthetase (WARS) | Rabbit | EPR3423 | Abcam, Cambridge, UK |
| Heat shock 70kDa protein 1-like (HSPA1L) | Rabbit |  | GeneTex, Irvine, CA |
| Leucine aminopeptidase 3 (LAP3) | Rabbit | EPR10330 | Abcam, Cambridge, UK |
| Apolipoprotein L2 (APOL2) | Rabbit |  | LifeSpan Biosciences, Seattle, WA |
