# Supplementary material for: Antibody-Validated Proteins in Inflamed Islets of Fulminant Type 1 Diabetes Profiled by Laser-Capture Microdissection Followed by Mass Spectrometry
Source: PLoS One. 2014 Oct 16;9(10):e107664. doi: 10.1371/journal.pone.0107664 (PMC4199548; doi:10.1371/journal.pone.0107664)
Supplement: Table S2 — Proteins identified in both islets affected by fulminant type 1 diabetes and in non-diabetic control pancreatic islets. (DOCX) [file pone.0107664.s002.docx]

| **Supporting Information Table S2.** | | | | | |
| --- | --- | --- | --- | --- | --- |
|  | | | | | |
|  | Accession number | Entry name | Protein names | Gene names | Molecular Weight |
| 1 | P16233 | LIPP_HUMAN | Pancreatic triacylglycerol lipase | PNLIP | 51 kDa |
| 2 | Q9P2E9 | RRBP1_HUMAN | Ribosome-binding protein 1 | RRBP1 | 152 kDa |
| 3 | P15085 | CBPA1_HUMAN | Carboxypeptidase A1 | CPA1 | 47 kDa |
| 4 | P11021 | GRP78_HUMAN | 78 kDa glucose-regulated protein | HSPA5 | 72 kDa |
| 5 | P01275 | GLUC_HUMAN | Glucagon | GCG | 21 kDa |
| 6 | P15086 | CBPB1_HUMAN | Carboxypeptidase B | CPB1 | 47 kDa |
| 7 | P05787 | K2C8_HUMAN | Keratin, type II cytoskeletal 8 | KRT8 | 54 kDa |
| 8 | P04746 | AMYP_HUMAN | Pancreatic alpha-amylase | AMY2A | 58 kDa |
| 9 | P02768 | ALBU_HUMAN | Serum albumin | ALB | 69 kDa |
| 10 | P08670 | VIME_HUMAN | Vimentin | VIM | 54 kDa |
| 11 | P60709 | ACTB_HUMAN | Actin, cytoplasmic 1 | ACTB | 42 kDa |
| 12 | P12111 | CO6A3_HUMAN | Collagen alpha-3(VI) chain | COL6A3 | 344 kDa |
| 13 | P19835 | CEL_HUMAN | Bile salt-activated lipase | CEL | 79 kDa |
| 14 | P06576 | ATPB_HUMAN | ATP synthase subunit beta, mitochondrial | ATP5B | 57 kDa |
| 15 | P14625 | ENPL_HUMAN | Endoplasmin | HSP90B1 | 92 kDa |
| 16 | P05783 | K1C18_HUMAN | Keratin, type I cytoskeletal 18 | KRT18 | 48 kDa |
| 17 | P07237 | PDIA1_HUMAN | Protein disulfide-isomerase | P4HB | 57 kDa |
| 18 | P50440 | GATM_HUMAN | Glycine amidinotransferase, mitochondrial | GATM | 48 kDa |
| 19 | P02545 | LMNA_HUMAN | Prelamin-A/C | LMNA | 74 kDa |
| 20 | P04264 | K2C1_HUMAN | Keratin, type II cytoskeletal 1 | KRT1 | 66 kDa |

**(Continued)**

**Supplementary Table S2. (Continued)**

| 21 | P09525 | ANXA4_HUMAN | Annexin A4 | ANXA4 | 36 kDa |
| --- | --- | --- | --- | --- | --- |
| 22 | Q13087 | PDIA2_HUMAN | Protein disulfide-isomerase A2 | PDIA2 | 58 kDa |
| 23 | P68371 | TBB4B_HUMAN | Tubulin beta-4B chain | TUBB4B | 50 kDa |
| 24 | P68871 | HBB_HUMAN | Hemoglobin subunit beta | HBB | 16 kDa |
| 25 | P07477 | TRY1_HUMAN | Trypsin-1 | PRSS1 | 27 kDa |
| 26 | P09093 | CEL3A_HUMAN | Chymotrypsin-like elastase family member 3A | CELA3A | 29 kDa |
| 27 | P30101 | PDIA3_HUMAN | Protein disulfide-isomerase A3 | PDIA3 | 57 kDa |
| 28 | P14618 | KPYM_HUMAN | Pyruvate kinase isozymes M1/M2 | PKM2 | 58 kDa |
| 29 | P68104 | EF1A1_HUMAN | Elongation factor 1-alpha 1 | EEF1A1 | 50 kDa |
| 30 | P08758 | ANXA5_HUMAN | Annexin A5 | ANXA5 | 36 kDa |
| 31 | P25705 | ATPA_HUMAN | ATP synthase subunit alpha, mitochondrial | ATP5A1 | 60 kDa |
| 32 | P10809 | CH60_HUMAN | 60 kDa heat shock protein, mitochondrial | HSPD1 | 61 kDa |
| 33 | P62258 | 1433E_HUMAN | 14-3-3 protein epsilon | YWHAE | 29 kDa |
| 34 | P07900 | HS90A_HUMAN | Heat shock protein HSP 90-alpha | HSP90AA1 | 85 kDa |
| 35 | P35579 | MYH9_HUMAN | Myosin-9 | MYH9 | 227 kDa |
| 36 | Q6GPI1 | CTRB2_HUMAN | Chymotrypsinogen B2 | CTRB2 | 28 kDa |
| 37 | P10645 | CMGA_HUMAN | Chromogranin-A | CHGA | 51 kDa |
| 38 | P22626 | ROA2_HUMAN | Heterogeneous nuclear ribonucleoproteins A2/B1 | HNRNPA2B1 | 37 kDa |
| 39 | O76038 | SEGN_HUMAN | Secretagogin | SCGN | 32 kDa |
| 40 | P08133 | ANXA6_HUMAN | Annexin A6 | ANXA6 | 76 kDa |
| 41 | P48052 | CBPA2_HUMAN | Carboxypeptidase A2 | CPA2 | 47 kDa |
| 42 | P62805 | H4_HUMAN | Histone H4 O | HIST1H4A | 11 kDa |
| 43 | P00352 | AL1A1_HUMAN | Retinal dehydrogenase 1 | ALDH1A1 | 55 kDa |
| 44 | P61978 | HNRPK_HUMAN | Heterogeneous nuclear ribonucleoprotein K | HNRNPK | 51 kDa |
| 45 | P06733 | ENOA_HUMAN | Alpha-enolase | ENO1 | 47 kDa |

**(Continued)**

**Supplementary Table S2. (Continued)**

| 46 | P55072 | TERA_HUMAN | Transitional endoplasmic reticulum ATPase | VCP | 89 kDa |
| --- | --- | --- | --- | --- | --- |
| 47 | Q15084 | PDIA6_HUMAN | Protein disulfide-isomerase A6 | PDIA6 | 48 kDa |
| 48 | P07355 | ANXA2_HUMAN | Annexin A2 | ANXA2 | 39 kDa |
| 49 | Q00610 | CLH1_HUMAN | Clathrin heavy chain 1 | CLTC | 192 kDa |
| 50 | P04406 | G3P_HUMAN | Glyceraldehyde-3-phosphate dehydrogenase | GAPDH | 36 kDa |
| 51 | P10909 | CLUS_HUMAN | Clusterin | CLU | 52 kDa |
| 52 | P29401 | TKT_HUMAN | Transketolase | TKT | 68 kDa |
| 53 | P69905 | HBA_HUMAN | Hemoglobin subunit alpha | HBA1 | 15 kDa |
| 54 | P11142 | HSP7C_HUMAN | Heat shock cognate 71 kDa protein | HSPA8 | 71 kDa |
| 55 | P06899 | H2B1J_HUMAN | Histone H2B type 1-J | HIST1H2BJ | 14 kDa |
| 56 | P40926 | MDHM_HUMAN | Malate dehydrogenase, mitochondrial | MDH2 | 36 kDa |
| 57 | P60174 | TPIS_HUMAN | Triosephosphate isomerase | TPI1 | 31 kDa |
| 58 | P63104 | 1433Z_HUMAN | 14-3-3 protein zeta/delta | YWHAZ | 28 kDa |
| 59 | P07478 | TRY2_HUMAN | Trypsin-2 | PRSS2 | 26 kDa |
| 60 | P27824 | CALX_HUMAN | Calnexin | CANX | 68 kDa |
| 61 | P32119 | PRDX2_HUMAN | Peroxiredoxin-2 | PRDX2 | 22 kDa |
| 62 | P26373 | RL13_HUMAN | 60S ribosomal protein L13 | RPL13 | 24 kDa |
| 63 | P80404 | GABT_HUMAN | 4-aminobutyrate aminotransferase, mitochondrial | ABAT | 56 kDa |
| 64 | P46781 | RS9_HUMAN | 40S ribosomal protein S9 | RPS9 | 23 kDa |
| 65 | P23396 | RS3_HUMAN | 40S ribosomal protein S3 | RPS3 | 27 kDa |
| 66 | P35527 | K1C9_HUMAN | Keratin, type I cytoskeletal 9 | KRT9 | 62 kDa |
| 67 | P02766 | TTHY_HUMAN | Transthyretin | TTR | 16 kDa |
| 68 | P12109 | CO6A1_HUMAN | Collagen alpha-1(VI) chain | COL6A1 | 109 kDa |
| 69 | Q13162 | PRDX4_HUMAN | Peroxiredoxin-4 | PRDX4 | 31 kDa |
| 70 | Q9UHG2 | PCSK1_HUMAN | ProSAAS | PCSK1N | 27 kDa |

**(Continued)**

**Supplementary Table S2. (Continued)**

| 71 | O43707 | ACTN4_HUMAN | Alpha-actinin-4 | ACTN4 | 105 kDa |
| --- | --- | --- | --- | --- | --- |
| 72 | P24752 | THIL_HUMAN | Acetyl-CoA acetyltransferase, mitochondrial | ACAT1 | 45 kDa |
| 73 | P05451 | REG1A_HUMAN | Lithostathine-1-alpha | REG1A | 19 kDa |
| 74 | P04843 | RPN1_HUMAN | Dolichyl-diphosphooligosaccharide--protein glycosyltransferase subunit 1 | RPN1 | 69 kDa |
| 75 | P07195 | LDHB_HUMAN | L-lactate dehydrogenase B chain | LDHB | 37 kDa |
| 76 | P53396 | ACLY_HUMAN | ATP-citrate synthase | ACLY | 121 kDa |
| 77 | P38646 | GRP75_HUMAN | Stress-70 protein, mitochondrial | HSPA9 | 74 kDa |
| 78 | O75396 | SC22B_HUMAN | Vesicle-trafficking protein SEC22b | SEC22B | 25 kDa |
| 79 | P04908 | H2A1B_HUMAN | Histone H2A type 1-B/E | HIST1H2AB | 14 kDa |
| 80 | P22314 | UBA1_HUMAN | Ubiquitin-like modifier-activating enzyme 1 | UBA1 | 118 kDa |
| 81 | P08865 | RSSA_HUMAN | 40S ribosomal protein SA | RPSA | 33 kDa |
| 82 | P46977 | STT3A_HUMAN | Dolichyl-diphosphooligosaccharide--protein glycosyltransferase subunit STT3A | STT3A | 81 kDa |
| 83 | P36578 | RL4_HUMAN | 60S ribosomal protein L4 | RPL4 | 48 kDa |
| 84 | Q9Y4L1 | HYOU1_HUMAN | Hypoxia up-regulated protein 1 | HYOU1 | 111 kDa |
| 85 | P04792 | HSPB1_HUMAN | Heat shock protein beta-1 | HSPB1 | 23 kDa |
| 86 | P02452 | CO1A1_HUMAN | Collagen alpha-1(I) chain | COL1A1 | 139 kDa |
| 87 | P07437 | TBB5_HUMAN | Tubulin beta chain | TUBB | 50 kDa |
| 88 | P48735 | IDHP_HUMAN | Isocitrate dehydrogenase [NADP], mitochondrial | IDH2 | 51 kDa |
| 89 | P55259 | GP2_HUMAN | Pancreatic secretory granule membrane major glycoprotein GP2 | GP2 | 59 kDa |
| 90 | P06753 | TPM3_HUMAN | Tropomyosin alpha-3 chain | TPM3 | 33 kDa |
| 91 | P23284 | PPIB_HUMAN | Peptidyl-prolyl cis-trans isomerase B | PPIB | 24 kDa |
| 92 | P05388 | RLA0_HUMAN | 60S acidic ribosomal protein P0 | RPLP0 | 34 kDa |
| 93 | P07910 | HNRPC_HUMAN | Heterogeneous nuclear ribonucleoproteins C1/C2 | HNRNPC | 34 kDa |
| 94 | P61204 | ARF3_HUMAN | ADP-ribosylation factor 3 | ARF3 | 21 kDa |
| 95 | P46783 | RS10_HUMAN | 40S ribosomal protein S10 | RPS10 | 19 kDa |

**(Continued)**

**Supplementary Table S2. (Continued)**

| 96 | P61278 | SMS_HUMAN | Somatostatin | SST | 13 kDa |
| --- | --- | --- | --- | --- | --- |
| 97 | P62269 | RS18_HUMAN | 40S ribosomal protein S18 | RPS18 | 18 kDa |
| 98 | P62820 | RAB1A_HUMAN | Ras-related protein Rab-1A | RAB1A | 23 kDa |
| 99 | P30044 | PRDX5_HUMAN | Peroxiredoxin-5, mitochondrial | PRDX5 | 22 kDa |
| 100 | P50395 | GDIB_HUMAN | Rab GDP dissociation inhibitor beta | GDI2 | 51 kDa |
| 101 | P49411 | EFTU_HUMAN | Elongation factor Tu, mitochondrial | TUFM | 50 kDa |
| 102 | P08263 | GSTA1_HUMAN | Glutathione S-transferase A1 | GSTA1 | 26 kDa |
| 103 | P30041 | PRDX6_HUMAN | Peroxiredoxin-6 | PRDX6 | 25 kDa |
| 104 | P51991 | ROA3_HUMAN | Heterogeneous nuclear ribonucleoprotein A3 | HNRNPA3 | 40 kDa |
| 105 | Q99623 | PHB2_HUMAN | Prohibitin-2 | PHB2 | 33 kDa |
| 106 | Q00341 | VIGLN_HUMAN | Vigilin | HDLBP | 141 kDa |
| 107 | P27797 | CALR_HUMAN | Calreticulin | CALR | 48 kDa |
| 108 | P00505 | AATM_HUMAN | Aspartate aminotransferase, mitochondrial | GOT2 | 48 kDa |
| 109 | P08238 | HS90B_HUMAN | Heat shock protein HSP 90-beta | HSP90AB1 | 83 kDa |
| 110 | Q03252 | LMNB2_HUMAN | Lamin-B2 | LMNB2 | 68 kDa |
| 111 | P39019 | RS19_HUMAN | 40S ribosomal protein S19 | RPS19 | 16 kDa |
| 112 | Q7KZF4 | SND1_HUMAN | Staphylococcal nuclease domain-containing protein 1 | SND1 | 102 kDa |
| 113 | P07737 | PROF1_HUMAN | Profilin-1 | PFN1 | 15 kDa |
| 114 | P15880 | RS2_HUMAN | 40S ribosomal protein S2 | RPS2 | 31 kDa |
| 115 | P62857 | RS28_HUMAN | 40S ribosomal protein S28 OS=Homo sapiens GN=RPS28 PE=1 SV=1 | RPS28 | 8 kDa |
| 116 | P09651 | ROA1_HUMAN | Heterogeneous nuclear ribonucleoprotein A1 | HNRNPA1 | 39 kDa |
| 117 | P14550 | AK1A1_HUMAN | Alcohol dehydrogenase [NADP(+)] | AKR1A1 | 37 kDa |
| 118 | P26641 | EF1G_HUMAN | Elongation factor 1-gamma | EEF1G | 50 kDa |
| 119 | P62753 | RS6_HUMAN | 40S ribosomal protein S6 | RPS6 | 29 kDa |
| 120 | P40313 | CTRL_HUMAN | Chymotrypsin-like protease CTRL-1 | CTRL | 28 kDa |

**(Continued)**

**Supplementary Table S2. (Continued)**

| 121 | P06744 | G6PI_HUMAN | Glucose-6-phosphate isomerase | GPI | 63 kDa |
| --- | --- | --- | --- | --- | --- |
| 122 | P0CG47 | UBB_HUMAN | Polyubiquitin-B | UBB | 26 kDa |
| 123 | P62913 | RL11_HUMAN | 60S ribosomal protein L11 | RPL11 | 20 kDa |
| 124 | P19338 | NUCL_HUMAN | Nucleolin | NCL | 77 kDa |
| 125 | P62081 | RS7_HUMAN | 40S ribosomal protein S7 | RPS7 | 22 kDa |
| 126 | P39656 | OST48_HUMAN | Dolichyl-diphosphooligosaccharide--protein glycosyltransferase 48 kDa subunit | DDOST | 51 kDa |
| 127 | P37802 | TAGL2_HUMAN | Transgelin-2 | TAGLN2 | 22 kDa |
| 128 | Q96KP4 | CNDP2_HUMAN | Cytosolic non-specific dipeptidase | CNDP2 | 53 kDa |
| 129 | P05387 | RLA2_HUMAN | 60S acidic ribosomal protein P2 | RPLP2 | 12 kDa |
| 130 | P60660 | MYL6_HUMAN | Myosin light polypeptide 6 | MYL6 | 17 kDa |
| 131 | P08727 | K1C19_HUMAN | Keratin, type I cytoskeletal 19 | KRT19 | 44 kDa |
| 132 | P08123 | CO1A2_HUMAN | Collagen alpha-2(I) chain | COL1A2 | 129 kDa |
| 133 | P62263 | RS14_HUMAN | 40S ribosomal protein S14 | RPS14 | 16 kDa |
| 134 | Q96DN0 | ERP27_HUMAN | Endoplasmic reticulum resident protein 27 | ERP27 | 30 kDa |
| 135 | P35232 | PHB_HUMAN | Prohibitin | PHB | 30 kDa |
| 136 | P98160 | PGBM_HUMAN | Basement membrane-specific heparan sulfate proteoglycan core protein | HSPG2 | 469 kDa |
| 137 | P62899 | RL31_HUMAN | 60S ribosomal protein L31 | RPL31 | 14 kDa |
| 138 | P62424 | RL7A_HUMAN | 60S ribosomal protein L7a | RPL7A | 30 kDa |
| 139 | P23246 | SFPQ_HUMAN | Splicing factor, proline- and glutamine-rich | SFPQ | 76 kDa |
| 140 | P61247 | RS3A_HUMAN | 40S ribosomal protein S3a | RPS3A | 30 kDa |
| 141 | P68431 | H31_HUMAN | Histone H3.1 | HIST1H3A | 15 kDa |
| 142 | Q00839 | HNRPU_HUMAN | Heterogeneous nuclear ribonucleoprotein U | HNRNPU | 91 kDa |
| 143 | Q13813 | SPTA2_HUMAN | Spectrin alpha chain, brain | SPTAN1 | 285 kDa |
| 144 | P30740 | ILEU_HUMAN | Leukocyte elastase inhibitor | SERPINB1 | 43 kDa |
| 145 | P62244 | RS15A_HUMAN | 40S ribosomal protein S15a | RPS15A | 15 kDa |

**(Continued)**

**Supplementary Table S2. (Continued)**

| 146 | P49368 | TCPG_HUMAN | T-complex protein 1 subunit gamma | CCT3 | 61 kDa |
| --- | --- | --- | --- | --- | --- |
| 147 | P35268 | RL22_HUMAN | 60S ribosomal protein L22 | RPL22 | 15 kDa |
| 148 | P49748 | ACADV_HUMAN | Very long-chain specific acyl-CoA dehydrogenase, mitochondrial | ACADVL | 70 kDa |
| 149 | P52565 | GDIR1_HUMAN | Rho GDP-dissociation inhibitor 1 | ARHGDIA | 23 kDa |
| 150 | Q08211 | DHX9_HUMAN | ATP-dependent RNA helicase A | DHX9 | 141 kDa |
| 151 | P36871 | PGM1_HUMAN | Phosphoglucomutase-1 | PGM1 | 61 kDa |
| 152 | P29692 | EF1D_HUMAN | Elongation factor 1-delta | EEF1D | 31 kDa |
| 153 | P13010 | XRCC5_HUMAN | X-ray repair cross-complementing protein 5 | XRCC5 | 83 kDa |
| 154 | Q86VP6 | CAND1_HUMAN | Cullin-associated NEDD8-dissociated protein 1 | CAND1 | 136 kDa |
| 155 | P04083 | ANXA1_HUMAN | Annexin A1 | ANXA1 | 39 kDa |
