# Supplementary material for: Antibody-Validated Proteins in Inflamed Islets of Fulminant Type 1 Diabetes Profiled by Laser-Capture Microdissection Followed by Mass Spectrometry
Source: PLoS One. 2014 Oct 16;9(10):e107664. doi: 10.1371/journal.pone.0107664 (PMC4199548; doi:10.1371/journal.pone.0107664)
Supplement: Table S3 — Proteins identified only in non-diabetic control pancreatic islets. (DOCX) [file pone.0107664.s003.docx]

| **Supporting Information Table S3.** | | | | | |
| --- | --- | --- | --- | --- | --- |
|  | | | | | |
|  | Accession number | Entry name | Protein names | Gene names | Molecular Weight |
| 1 | P68363 | TBA1B_HUMAN | Tubulin alpha-1B chain | TUBA1B | 50 kDa |
| 2 | P13645 | K1C10_HUMAN | Keratin, type I cytoskeletal 10 | KRT10 | 59 kDa |
| 3 | P13639 | EF2_HUMAN | Elongation factor 2 | EEF2 | 95 kDa |
| 4 | P06899 | H2B1J_HUMAN | Histone H2B type 1-J | HIST1H2BJ | 14 kDa |
| 5 | P00558 | PGK1_HUMAN | Phosphoglycerate kinase 1 | PGK1 | 45 kDa |
| 6 | Q99895 | CTRC_HUMAN | Chymotrypsin-C | CTRC | 29 kDa |
| 7 | P30086 | PEBP1_HUMAN | Phosphatidylethanolamine-binding protein 1 | PEBP1 | 21 kDa |
| 8 | P16870 | CBPE_HUMAN | Carboxypeptidase E | CPE | 53 kDa |
| 9 | Q02878 | RL6_HUMAN | 60S ribosomal protein L6 | RPL6 | 33 kDa |
| 10 | O43175 | SERA_HUMAN | D-3-phosphoglycerate dehydrogenase | PHGDH | 57 kDa |
| 11 | P54317 | LIPR2_HUMAN | Pancreatic lipase-related protein 2 | PNLIPRP2 | 52 kDa |
| 12 | P12110 | CO6A2_HUMAN | Collagen alpha-2(VI) chain | COL6A2 | 109 kDa |
| 13 | P05060 | SCG1_HUMAN | Secretogranin-1 | CHGB | 78 kDa |
| 14 | P09211 | GSTP1_HUMAN | Glutathione S-transferase P | GSTP1 | 23 kDa |
| 15 | P07099 | HYEP_HUMAN | Epoxide hydrolase 1 | EPHX1 | 53 kDa |
| 16 | P62937 | PPIA_HUMAN | Peptidyl-prolyl cis-trans isomerase A | PPIA | 18 kDa |
| 17 | P08217 | CEL2A_HUMAN | Chymotrypsin-like elastase family member 2A | CELA2A | 29 kDa |
| 18 | P08861 | CEL3B_HUMAN | Chymotrypsin-like elastase family member 3B | CELA3B | 29 kDa |
| 19 | P04075 | ALDOA_HUMAN | Fructose-bisphosphate aldolase A | ALDOA | 39 kDa |
| 20 | P46782 | RS5_HUMAN | 40S ribosomal protein S5 | RPS5 | 23 kDa |

**(Continued)**

**Supplementary Table 3. (Continued)**

| 21 | P35908 | K22E_HUMAN | Keratin, type II cytoskeletal 2 epidermal | KRT2 | 65 kDa |
| --- | --- | --- | --- | --- | --- |
| 22 | P27348 | 1433T_HUMAN | 14-3-3 protein theta | YWHAQ | 28 kDa |
| 23 | P62158 | CALM_HUMAN | Calmodulin | CALM1 | 17 kDa |
| 24 | P08107 | HSP71_HUMAN | Heat shock 70 kDa protein 1A/1B | HSPA1A | 70 kDa |
| 25 | P04118 | COL_HUMAN | Colipase | CLPS | 12 kDa |
| 26 | Q16836 | HCDH_HUMAN | Hydroxyacyl-coenzyme A dehydrogenase, mitochondrial HADH | HADH | 34 kDa |
| 27 | P55084 | ECHB_HUMAN | Trifunctional enzyme subunit beta, mitochondrial | HADHB | 51 kDa |
| 28 | P63244 | GBLP_HUMAN | Guanine nucleotide-binding protein subunit beta-2-like 1 | GNB2L1 | 35 kDa |
| 29 | P04844 | RPN2_HUMAN | Dolichyl-diphosphooligosaccharide--protein glycosyltransferase subunit 2 | RPN2 | 69 kDa |
| 30 | Q14103 | HNRPD_HUMAN | Heterogeneous nuclear ribonucleoprotein D0 | HNRNPD | 38 kDa |
| 31 | Q96AG4 | LRC59_HUMAN | Leucine-rich repeat-containing protein 59 | LRRC59 | 35 kDa |
| 32 | P62701 | RS4X_HUMAN | 40S ribosomal protein S4, X isoform | RPS4X | 30 kDa |
| 33 | O43390 | HNRPR_HUMAN | Heterogeneous nuclear ribonucleoprotein R | HNRNPR | 71 kDa |
| 34 | Q14240 | IF4A2_HUMAN | Eukaryotic initiation factor 4A-II OS=Homo sapiens GN=EIF4A2 PE=1 SV=2 | EIF4A2 | 46 kDa |
| 35 | P14174 | MIF_HUMAN | Macrophage migration inhibitory factor | MIF | 12 kDa |
| 36 | P19961 | AMY2B_HUMAN | Alpha-amylase 2B | AMY2B | 58 kDa |
| 37 | P18124 | RL7_HUMAN | 60S ribosomal protein L7 | RPL7 | 29 kDa |
| 38 | P62241 | RS8_HUMAN | 40S ribosomal protein S8 | RPS8 | 24 kDa |
| 39 | Q99798 | ACON_HUMAN | Aconitate hydratase, mitochondrial | ACO2 | 85 kDa |
| 40 | P05141 | ADT2_HUMAN | ADP/ATP translocase 2 | SLC25A5 | 33 kDa |
| 41 | P61604 | CH10_HUMAN | 10 kDa heat shock protein, mitochondrial | HSPE1 | 11 kDa |
| 42 | P53621 | COPA_HUMAN | Coatomer subunit alpha O | COPA | 138 kDa |
| 43 | P00367 | DHE3_HUMAN | Glutamate dehydrogenase 1, mitochondrial | GLUD1 | 61 kDa |
| 44 | Q9NYL4 | FKB11_HUMAN | Peptidyl-prolyl cis-trans isomerase FKBP11 | FKBP11 | 22 kDa |
| 45 | P83731 | RL24_HUMAN | 60S ribosomal protein L24 | RPL24 | 18 kDa |

**(Continued)**

**Supplementary Table 3. (Continued)**

| 46 | P62277 | RS13_HUMAN | 40S ribosomal protein S13 | RPS13 | 17 kDa |
| --- | --- | --- | --- | --- | --- |
| 47 | P00338 | LDHA_HUMAN | L-lactate dehydrogenase A chain | LDHA | 37 kDa |
| 48 | P05023 | AT1A1_HUMAN | Sodium/potassium-transporting ATPase subunit alpha-1 | ATP1A1 | 113 kDa |
| 49 | O75367 | H2AY_HUMAN | Core histone macro-H2A.1 | H2AFY | 40 kDa |
| 50 | P01308 | INS_HUMAN | Insulin | INS | 12 kDa |
| 51 | P61224 | RAP1B_HUMAN | Ras-related protein Rap-1b | RAP1B | 21 kDa |
| 52 | Q02543 | RL18A_HUMAN | 60S ribosomal protein L18a | RPL18A | 21 kDa |
| 53 | P13521 | SCG2_HUMAN | Secretogranin-2 | SCG2 | 71 kDa |
| 54 | P40939 | ECHA_HUMAN | Trifunctional enzyme subunit alpha, mitochondrial | HADHA | 83 kDa |
| 55 | P18085 | ARF4_HUMAN | ADP-ribosylation factor 4 | ARF4 | 21 kDa |
| 77 | P68366 | TBA4A_HUMAN | Tubulin alpha-4A chain | TUBA4A | 50 kDa |
| 78 | P84103 | SRSF3_HUMAN | Serine/arginine-rich splicing factor 3 | SRSF3 | 19 kDa |
| 79 | O43598 | RCL_HUMAN | Deoxyribonucleoside 5'-monophosphate N-glycosidase | RCL | 19 kDa |
| 80 | Q14749 | GNMT_HUMAN | Glycine N-methyltransferase | GNMT | 33 kDa |
| 81 | Q16698 | DECR_HUMAN | 2,4-dienoyl-CoA reductase, mitochondrial | DECR1 | 36 kDa |
| 82 | Q14011 | CIRBP_HUMAN | Cold-inducible RNA-binding protein | CIRBP | 19 kDa |
| 83 | P16152 | CBR1_HUMAN | Carbonyl reductase [NADPH] 1 | CBR1 | 30 kDa |
| 84 | P24534 | EF1B_HUMAN | Elongation factor 1-beta | EEF1B2 | 25 kDa |
| 85 | O15240 | VGF_HUMAN | Neurosecretory protein VGF | VGF | 67 kDa |
| 86 | Q13724 | MOGS_HUMAN | Mannosyl-oligosaccharide glucosidase | MOGS | 92 kDa |
| 87 | Q9Y678 | COPG1_HUMAN | Coatomer subunit gamma-1 | COPG1 | 98 kDa |
| 88 | P54886 | P5CS_HUMAN | Delta-1-pyrroline-5-carboxylate synthase | ALDH18A1 | 87 kDa |
| 89 | P39023 | RL3_HUMAN | 60S ribosomal protein L3 | RPL3 | 46 kDa |
| 90 | Q0VAF6 | SYCN_HUMAN | Syncollin | YCN | 14 kDa |

**(Continued)**

**Supplementary Table 3. (Continued)**

| 91 | Q9BRL6 | SRSF8_HUMAN | Serine/arginine-rich splicing factor 8 | SRSF8 | 32 kDa |
| --- | --- | --- | --- | --- | --- |
| 92 | P30048 | PRDX3_HUMAN | Thioredoxin-dependent peroxide reductase, mitochondrial | PRDX3 | 28 kDa |
| 93 | P00403 | COX2_HUMAN | Cytochrome c oxidase subunit 2 | MT-CO2 | 26 kDa |
| 94 | P01034 | CYTC_HUMAN | Cystatin-C | CST3 | 16 kDa |
| 95 | P26640 | SYVC_HUMAN | Valine--tRNA ligase | VARS | 140 kDa |
| 96 | P06396 | GELS_HUMAN | Gelsolin | GSN | 86 kDa |
| 97 | P43243 | MATR3_HUMAN | Matrin-3 | MATR3 | 95 kDa |
| 98 | Q02252 | MMSA_HUMAN | Methylmalonate-semialdehyde dehydrogenase [acylating], mitochondrial | ALDH6A1 | 58 kDa |
| 99 | Q07065 | CKAP4_HUMAN | Cytoskeleton-associated protein 4 | CKAP4 | 66 kDa |
| 100 | P55327 | TPD52_HUMAN | Tumor protein D52 | TPD52 | 24 kDa |
| 101 | P39060 | COIA1_HUMAN | Collagen alpha-1(XVIII) chain | COL18A1 | 178 kDa |
| 102 | P21399 | ACOC_HUMAN | Cytoplasmic aconitate hydratase | ACO1 | 98 kDa |
| 103 | P09622 | DLDH_HUMAN | Dihydrolipoyl dehydrogenase, mitochondrial | DLD | 54 kDa |
| 104 | P21796 | VDAC1_HUMAN | Voltage-dependent anion-selective channel protein 1 | VDAC1 | 31 kDa |
| 105 | P36542 | ATPG_HUMAN | ATP synthase subunit gamma, mitochondrial | ATP5C1 | 33 kDa |
| 106 | P50991 | TCPD_HUMAN | T-complex protein 1 subunit delta | CCT4 | 58 kDa |
| 107 | P13804 | ETFA_HUMAN | Electron transfer flavoprotein subunit alpha, mitochondrial | ETFA | 35 kDa |
